# New nanostructured silica incorporated with isolated Ti material for the photocatalytic conversion of CO_2_ to fuels

**DOI:** 10.1186/1556-276X-9-158

**Published:** 2014-04-01

**Authors:** Parveen Akhter, Murid Hussain, Guido Saracco, Nunzio Russo

**Affiliations:** 1 Department of Applied Science and Technology, Politecnico di Torino, Corso Duca degli Abruzzi 24, Torino 10129, Italy; 2 Department of Chemical Engineering, COMSATS Institute of Information Technology, M A Jinnah Building, Defence Road, Off Raiwind Road, Lahore 54000, Pakistan

**Keywords:** Isolated Ti, Carbon dioxide, Water vapors, Fuels, Photocatalysis

## Abstract

In this work, new nanoporous silica (Korea Advanced Institute of Science and Technology-6 (KIT-6)-dried or KIT-6-calcined) incorporated with isolated Ti materials with different Si/Ti ratios (Si/Ti = 200, 100, and 50) has been synthesized and investigated to establish photocatalytic reduction of CO_2_ in the presence of H_2_O vapors. The properties of the materials have been characterized through N_2_ adsorption/desorption, UV-vis, TEM, FT-IR, and XPS analysis techniques. The intermediate amount of the isolated Ti (Si/Ti = 100) has resulted to be more uniformly distributed on the surface and within the three-dimensional pore structure of the KIT-6 material, without its structure collapsing, than the other two ratios (Si/Ti = 200 and 50). However, titania agglomerates have been observed to have formed due to the increased Ti content (Si/Ti = 50). The Ti-KIT-6 (calcined) materials in the reaction showed higher activity than the Ti-KIT-6 (dried) materials, which produced CH_4_, H_2_, CO, and CH_3_OH (vapors) as fuel products. The Ti-KIT-6 (Si/Ti = 100) material also showed more OH groups, which are useful to obtain a higher production rate of the products, particularly methane, which was even higher than the rate of the best commercial TiO_2_ (Aeroxide P25, Evonik Industries AG, Essen, Germany) photocatalyst.

## Background

The gradual increase in the world population and the industrial development have both led to high energy consumption and the unabated release of toxic agents and industrial wastes into the air and waterways, which in turn have led to pollution-related diseases, global warming, and abnormal climatic changes [1]. Carbon dioxide (CO_2_), which is mainly obtained from fossil fuel combustion, plays a significant role in global heating [2] and is currently considered a key challenge for the world. At present, the most optimized and preferable way of reducing CO_2_ is to recycle it as a fuel feedstock, with energy input from cheap and abundant sources [3]. Moreover, due to the shortages and restrictions on the use of fossil fuels and the increased energy demand, there has been increasing interest in the development of alternative renewable energy resources, which has encouraged researchers to use CO_2_ as a raw material to produce fuels [1-4].

Photocatalytic CO_2_ reduction is highly popular but still in an embryonic stage. It simply uses ultraviolet (UV) and/or visible light as the excitation source for semiconductor catalysts. The photoexcited electrons reduce CO_2_ with H_2_O on the catalyst surface to form energy-bearing products, such as carbon monoxide (CO), methane (CH_4_), methanol (CH_3_OH), formaldehyde (HCHO), and formic acid (HCOOH) [1-4]. TiO_2_, CdS, ZrO_2_, ZnO, and MgO photocatalysts have been investigated in this context. However, wide-bandgap TiO_2_ photocatalysts are considered the most convenient candidates, in terms of cost and stability [5,6].

Recently, the design of highly efficient and selective photocatalytic systems for the reduction of CO_2_ with H_2_O vapors has been of key interest. It has been shown in the literature [7] that highly dispersed titanium oxide (Ti oxide) catalysts anchored on porous Vycor glass (Amsterdam, The Netherlands), zeolites, and some nanoporous silica materials, such as Mobil Composition of Matter-41 (MCM-41), show better photocatalytic activity for CO_2_ conversion than bulk TiO_2_ powder. However, MCM-41 mesoporous silica has a one-dimensional (1-D, hexagonal p6mm) pore structure, with a relatively small pore size and poor hydrothermal stability. Korea Advanced Institute of Science and Technology-6 (KIT-6) silica is another interesting alternative material to MCM-41. It has a three-dimensional (3-D) (gyroid cubic Ia3d) pore structure and large pore size and has recently received the attention of many researchers in various applications [8,9].

In the present work, a new KIT-6 mesoporous silica (with interesting physical properties, cavities, and frameworks) incorporated with highly dispersed isolated Ti materials has been synthesized; characterized by means of UV-visible (UV-vis) spectroscopy, transmission electron microscopy (TEM), Fourier transform infrared spectroscopy (FT-IR), and X-ray photoelectron spectroscopy (XPS) analysis techniques; and applied for the photocatalytic reduction of CO_2_ with H_2_O vapors to obtain fuels (CH_4_, H_2_, CO, and CH_3_OH). The activity results have been compared with the best commercial TiO_2_ photocatalyst (Aeroxide P25, Evonik Industries AG, Essen, Germany), and the involved mechanism has been discussed.

## Methods

### Synthesis of the materials

The mesoporous silica material (KIT-6) was obtained by following the procedure shown in recent works [8,9]. After a hydrothermal treatment, the obtained solid product was filtered, dried, and/or calcined at 550°C for 5 h and was then utilized to prepare Ti-KIT-6 (dried or calcined). The dried and calcined KIT-6 materials were then treated with titanium (IV) isopropoxide (98%) at different Si/Ti ratios (200, 100, and 50), and finally calcined to obtain Ti-KIT-6 according the procedure recently reported in [10].

### Characterization of the materials

The UV-vis diffuse reflectance spectra were recorded using a Varian model Cary 500 spectrophotometer with a quartz cell (Palo Alto, CA, USA) suitable for measuring powders. The Brunauer-Emmett-Teller (BET) specific surface area (*S*_BET_), pore volume (PV), and average pore diameter (APD) were measured on the powder materials, which had previously been outgassed at 150°C using Micromeritics FlowPrep 060 (Norcross, GA, USA) (sample degas system), by means of N_2_ sorption at 77 K on a Micromeritics Tristar II (surface area and porosity) instrument. The TEM images were taken from the thin edges of the sample particles using a TEM Philips CM12 (Amsterdam, Netherlands), with a LaB6 filament and a double-tilt holder, operating at 120 kV. The FT-IR spectra were collected at a resolution of 2 cm^−1^ on a PerkinElmer FT-IR spectrophotometer equipped with an MCT detector (Waltham, MA, USA). The XPS spectra were recorded using a PHI 5000 Versa Probe (Chanhassen, MN, USA), with a scanning ESCA microscope (Trieste, Italy) fitted with an Al monochromatic X-ray source (1486.6 eV, 25.6 W), a beam diameter of 100 μm, a neutralizer at 1.4 eV 20 mA, and in FAT analyzer mode.

### Photocatalytic reaction

The basic experimental setup can be found in the previous work [11]. It includes a Pyrex glass reactor (Savat di Rasetti Giuseppe & C. S.a.s, Torino, Italy), connectors, mass flow controllers, water bubbler, and a UV lamp (300 W, Osram Ultra-Vitalux, Munich, Germany). It also has a CO_2_ gas cylinder (99.99%), a gas chromatograph (Varian CP-3800) equipped with a capillary column (CP7381), a flame ionization detector (FID), and a thermal conductivity detector (TCD). A photocatalytic reaction was performed in the reactor, which contained 0.5 g of photocatalyst. CO_2_ gas was introduced into the reactor at 50 mL/min for 30 min, after passing it through the water bubbler and has an adsorption-desorption balance; this is to saturate the catalysts with CO_2_ and H_2_O. A 0.1-g glass wool wet with 0.5 mL of H_2_O was also placed in the reactor at the entrance point of the CO_2_ and H_2_O to balance the water deficiency in the reactor. After 30 min, the CO_2_ flow rate was reduced to 10 mL/min. When equilibrium was reached, the UV light was turned on, and the reaction products were analyzed by means of the GC. Blank tests were also conducted to ensure that the product was due to the photocatalytic reaction. The blank tests consisted of a UV illumination without the photocatalyst and a reaction in the dark with the catalyst.

## Results and discussion

### Physicochemical properties of the synthesized materials

Table 1 shows the physical and textural properties of the KIT-6 and Ti-KIT-6 materials, which were obtained by means of N_2_ sorption. A noticeable decrease can be seen in the surface area and pore volume of KIT-6, after Ti incorporation with different Si/Ti ratios. However, the surface area and pore volume of the Ti-KIT-6 (dried) materials were slightly higher than those of the Ti-KIT-6 (calcined) ones, which might be due to the easy incorporation of Ti in the dried weak structure of KIT-6. However, Ti can be trapped in the bulk of the dried KIT-6 material, but not in that of the rigid structure of the calcined KIT-6 one. The average pore diameter did not change significantly and remained uniform, which might be due to the 3-D pore structure of KIT-6, which is able to accommodate the uniform isolated Ti dispersion.

**Table 1:** Comparison of the physical properties, bandgap energy of the synthesized materials, and methane production

**Samples**	**N**_**2 **_**sorption**			**UV-vis**		**CH**_**4 **_**production comparison**	
	***S***_**BET**_	**PV**	**APD**	**WL**	**BE**	***P***	**Reference**
[Ti-K-6 (dried) (Si/Ti = 200)] calcined	865	1.11	6.55	-	-	-	-
[Ti-K-6 (dried) (Si/Ti = 100)] calcined	767	0.80	6.48	-	-	-	-
[Ti-K-6 (dried) (Si/Ti = 50)] calcined	730	0.67	6.45	-	-	-	-
KIT-6 (K-6) calcined	772	1.04	6.49	-	-	-	-
[Ti-K-6 (calcined) (Si/Ti = 200)] calcined	726	0.95	6.45	320	3.87	-	-
[Ti-K-6 (calcined) (Si/Ti = 100)] calcined	700	0.85	6.40	330	3.75	4.1	This work
[Ti-K-6 (calcined) (Si/Ti = 50)] calcined	684	0.73	6.41	372	3.33	-	-
Anatase TiO_2_ powder	-	-	-	-	-	0.4	[18]
Aeroxide/degussa P25 TiO_2_	-	-	-	-	-	0.6	This work
Titanium silicate (TS-1) zeolite	-	-	-	-	-	2.7	[16]
Ti-MCM-41	-	-	-	-	-	2.9	[16]

*S*_BET_, BET specific surface area in m^2^/g; PV, cumulative pore volume in cm^3^/g; APD, average pore diameter in nm; WL, absorption edge wave length, *λ*, in nm; BE, bandgap energy in eV; *P*, production rate in μmol · g_cat_.^−1^ · h^−1^).

**Figure 1:**
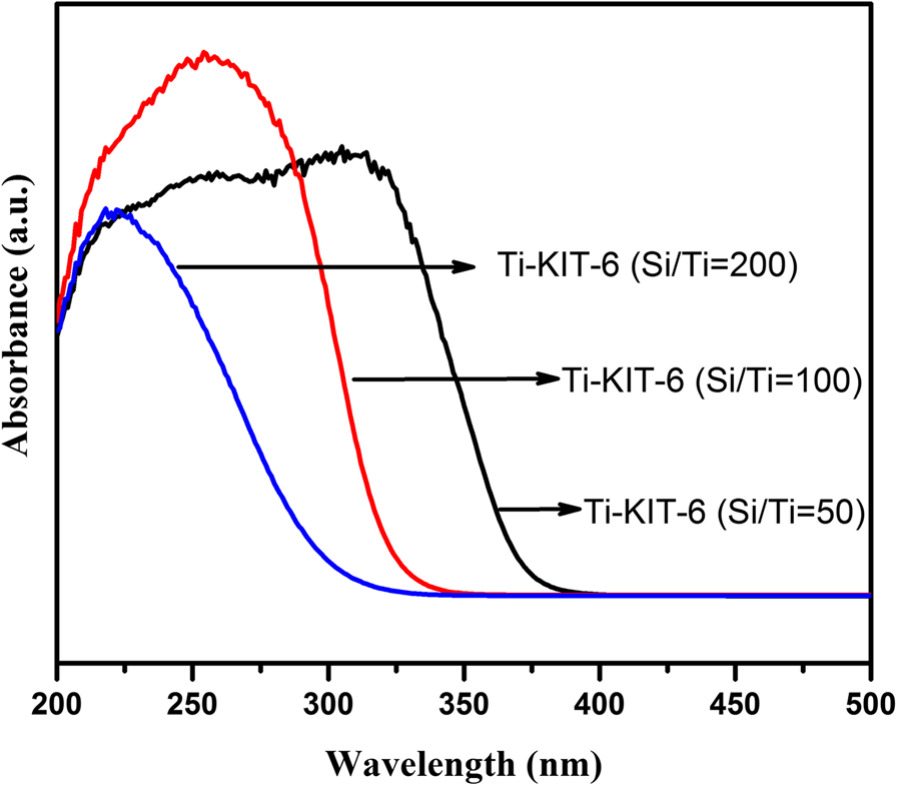
UV-vis spectra of the Ti-KIT-6 (calcined, Si/Ti = 200, 100, and 50 ratios) materials.

The UV-vis spectra of the calcinated Ti-KIT-6 (calcined, Si/Ti = 200, 100, and 50) are shown in Figure 1. It has been observed that with the increased Ti content, the absorption spectra are shifted to higher wavelengths since the absorption edge wavelength changes from 320 to 372 nm (Table 1), that is, moving towards the trend of pure TiO_2_. Therefore, it can be observed that this increased Ti might also have more chance of making the agglomerates of TiO_2_ with the moisture present during the synthesis. The bandgap energies of the Ti-KIT-6 materials, corresponding to a bandgap of 3.33 to 3.87 eV, which is the characteristic bandgap of Ti-silica, are shown in Table 1. The variation in the bandgap is due to the TiO_2_ agglomerates that have formed, as already mentioned, and which will be dealt with in more detail hereafter.

**Figure 2:**
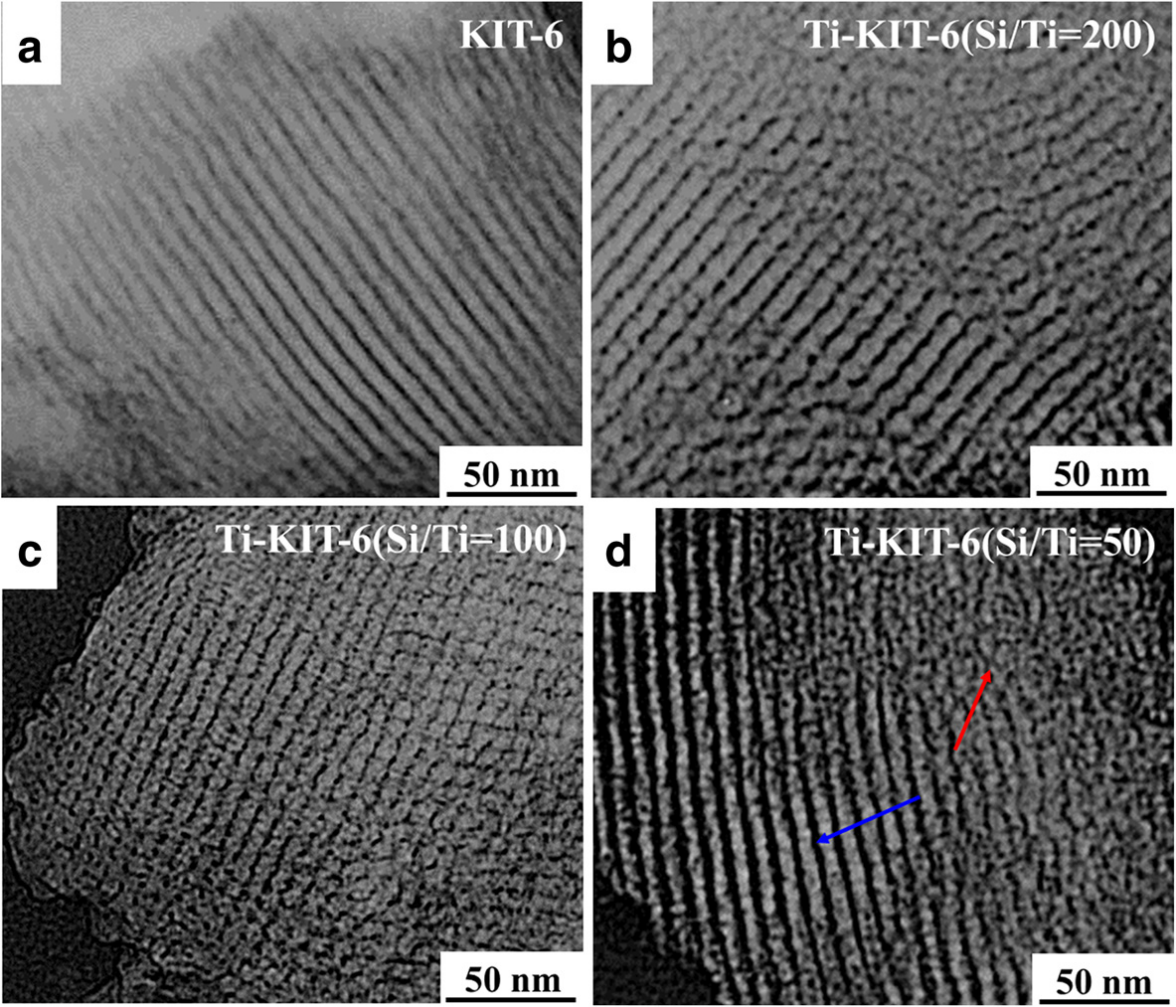
**TEM images. (a)** KIT-6 (calcined), **(b)** Ti-KIT-6 (calcined, Si/Ti = 200), **(c)** Ti-KIT-6 (calcined, Si/Ti = 100), and **(d)** Ti-KIT-6 (calcined, Si/Ti = 50). The blue arrow shows the preserved meso-structure. The red arrow indicates the partial collapse of the mesoporous structure.

**Figure 3:**
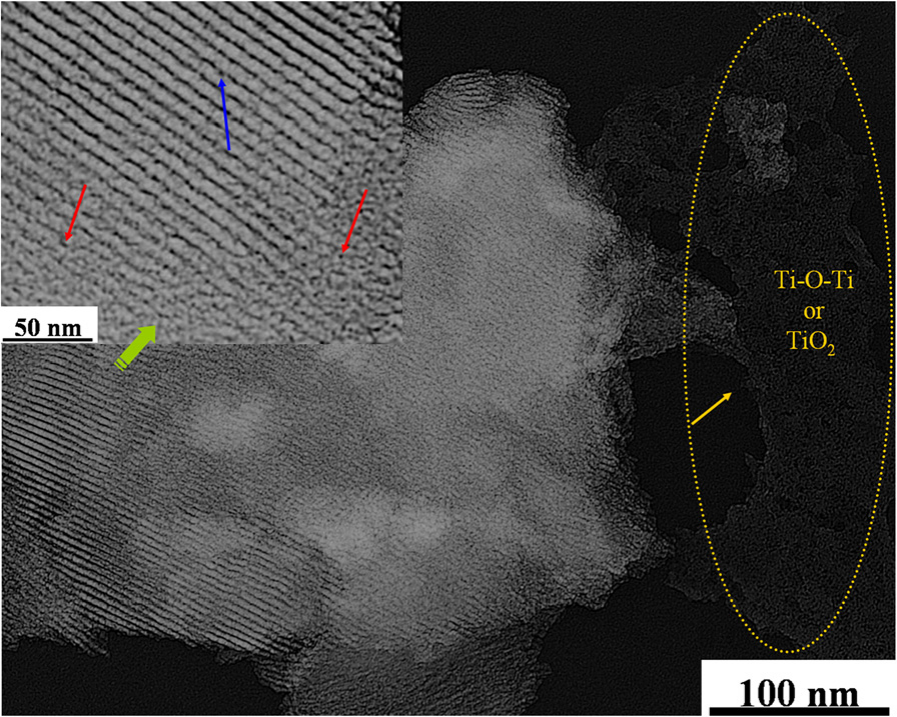
**TEM image of Ti-KIT-6 (calcined, Si/Ti = 50).** The image shows an overall view of the Ti distribution and TiO_2_ formation. The blue arrow shows the preserved meso-structure. The red arrow indicates the partial collapse of the mesoporous structure.

The TEM analysis pointed out a mesoporous structure in the KIT-6 material and isolated Ti dispersion within the KIT-6 structure. Figure 2a shows an ordered array of mesopores, which indicates the successful formation of the KIT-6 structure, where the centers of two adjacent pores are about 10 nm apart; a pore diameter of 6 nm can also be observed. This finding concerning APD is also in agreement with the result obtained from N_2_ sorption shown in Table 1 and that reported in the literature [9]. The TEM images of Ti-KIT-6 (Si/Ti ratios of 200, 100, and 50) are shown in Figure 2b,c,d. As shown in Figure 2b, Ti-KIT-6 (200) shows a uniform Ti dispersion with hardly any Ti agglomeration, which indicates the preserved structure of the support material, as is confirmed by the mesoporous channels of KIT-6. Ti-KIT-6 (100) has shown a similar trend to Ti-KIT-6 (200). A good dispersion of isolated Ti and mesopore structure preservation can be observed (Figure 2c). However, it can also be observed that the mesopore structure of KIT-6 is partially collapsed/damaged in Ti-KIT-6 (50) (see the right corner in Figure 2d), due to the higher Ti content than for the other two ratios. Figure 3, in which Ti dispersion and partial collapse of the mesopores of KIT-6 after Ti anchoring (Si/Ti = 50) is obvious, demonstrates this effect more clearly. However, despite the Ti isolated species being dispersed on the KIT-6 support material, some Ti-O-Ti or TiO_2_ agglomerates that were not observed in Ti-KIT-6 (200 and 100), but only in Ti-KIT-6 (50), have also been detected. This is due to the increased Ti which is not uniformly dispersed, and either forms Ti-O-Ti agglomerates or produces TiO_2_ due to the moisture.

**Figure 4:**
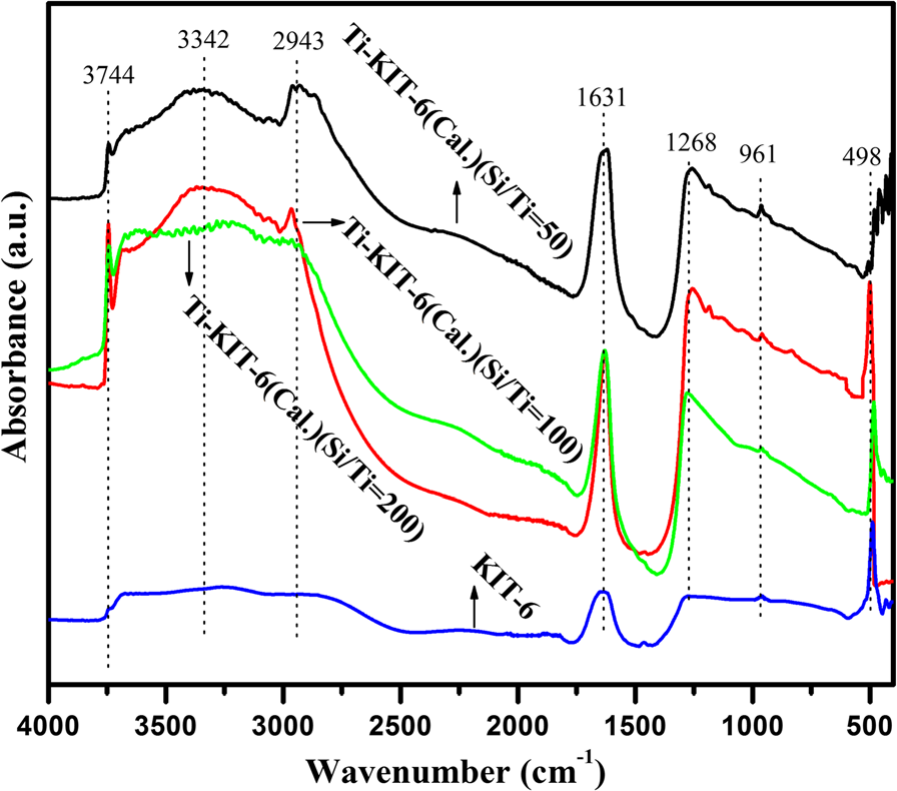
FT-IR analysis spectra of KIT-6 (calcined) and Ti-KIT-6 (calcined, Si/Ti = 200, 100, and 50 ratios) materials.

The FT-IR spectra of the KIT-6 and Ti-KIT-6 (200, 100, and 50) materials are shown in Figure 4. The bands that appeared at 498 and 1,268 cm^−1^ in the IR spectra for KIT-6 represent Si-O-Si [12]; the band at 1,631 cm^−1^ is due to the OH from the water occluded in the KIT-6 pores, whereas the band at 961 cm^−1^ is due to Si-OH. The central spectra at 3,342 cm^−1^ of the 3,100- to 3,600-cm^−1^ region can be ascribed to the hydroxyl groups from silanols as well as the OH of the adsorbed water. Moreover, the stretching observed at 3,742 cm^−1^ is due to the free OH groups [12,13]. The additional stretching in the Ti-KIT-6 material that appeared at 961 cm^−1^ is due to Ti-O-Si [12], in which Ti was attached through the hydroxyl groups of the KIT-6 silica. An increase in the peak intensity has been found for an increase in the Ti content for Si/Ti ratios of 200 to 50; this is generally considered as proof of Ti incorporation within the framework of KIT-6. Moreover, an additional stretching of Ti-O-Ti has been observed at 435 cm^−1^ due to the increased Ti content in Si/Ti = 50. Overall, the OH groups that represent the adsorption power of the material were also increased in the Ti-KIT-6 samples from Si/Ti ratios of 200 to 100, and then a slight decrease was found in the 50 ratio. This increase in OH groups might be associated with the better dispersion of the isolated Ti species on KIT-6 with Si/Ti = 100 than for the other ratios of 200 and 50, and it is also a sign of the good hydrophilicity of the material.

**Figure 5:**
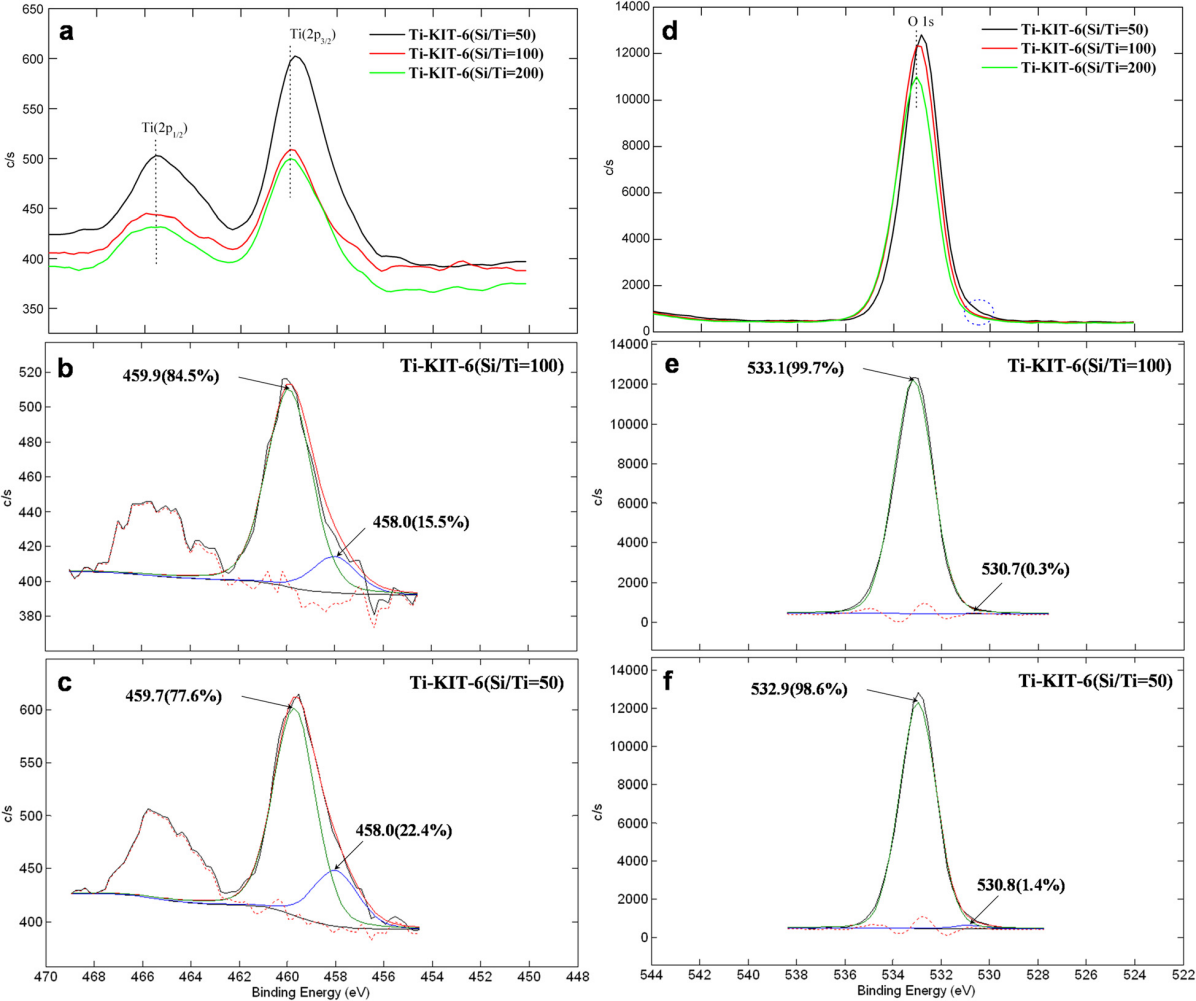
**XPS analysis of Ti-KIT-6 (calcined) materials showing the difference in the different samples. (a)** Overall Ti2*p* and **(b**,**c)** deconvolution of Ti-KIT-6 (calcined, Si/Ti = 100 and 50 ratios). **(d)** Overall O1s and **(e**,**f)** deconvolution of Ti-KIT-6 (calcined, Si/Ti = 100 and 50 ratios).

The Ti(2*p*) XPS spectra for Ti-KIT-6 are shown in Figure 5a, for different Ti contents, where a Ti(2*p*_3/2_) and Ti(2*p*_1/2_) doublet with a separation of 5.75 eV [14] can be seen. The Ti(2*p*_3/2_) line was shifted towards a lower binding energy for an increased Ti content of Si/Ti ratios of 200 to 50. The deconvoluted XPS spectra shown in Figure 5b,c indicates that for an increased Ti content of Si/Ti = 50, the Ti(2*p*_3/2_) line was shifted even further to 458.0 eV, which is close to the binding energy of Ti(2*p*_3/2_) of pure titania. As can be seen in Figure 5d,e,f, similar behavior has been noticed in the O1s spectra of the Ti-KIT-6 materials, in which the O1s line at 533 eV gradually shifted towards lower binding energies for an increased Ti content. The deconvoluted XPS spectra of Ti-KIT-6, at Si/Ti ratios of 100 and 50, depicted two peaks at 533 eV for Si-O-Si and 530.8 eV corresponding to Ti-O-Ti. These indicate that there is more free TiO_2_ phase formation in Ti-KIT-6(Si/Ti = 50) than in Ti-KIT-6(Si/Ti = 100). This is also in agreement with the results of the UV-vis and TEM analyses.

### Photocatalytic conversion of CO_2_ to fuels and its mechanism

**Figure 6:**
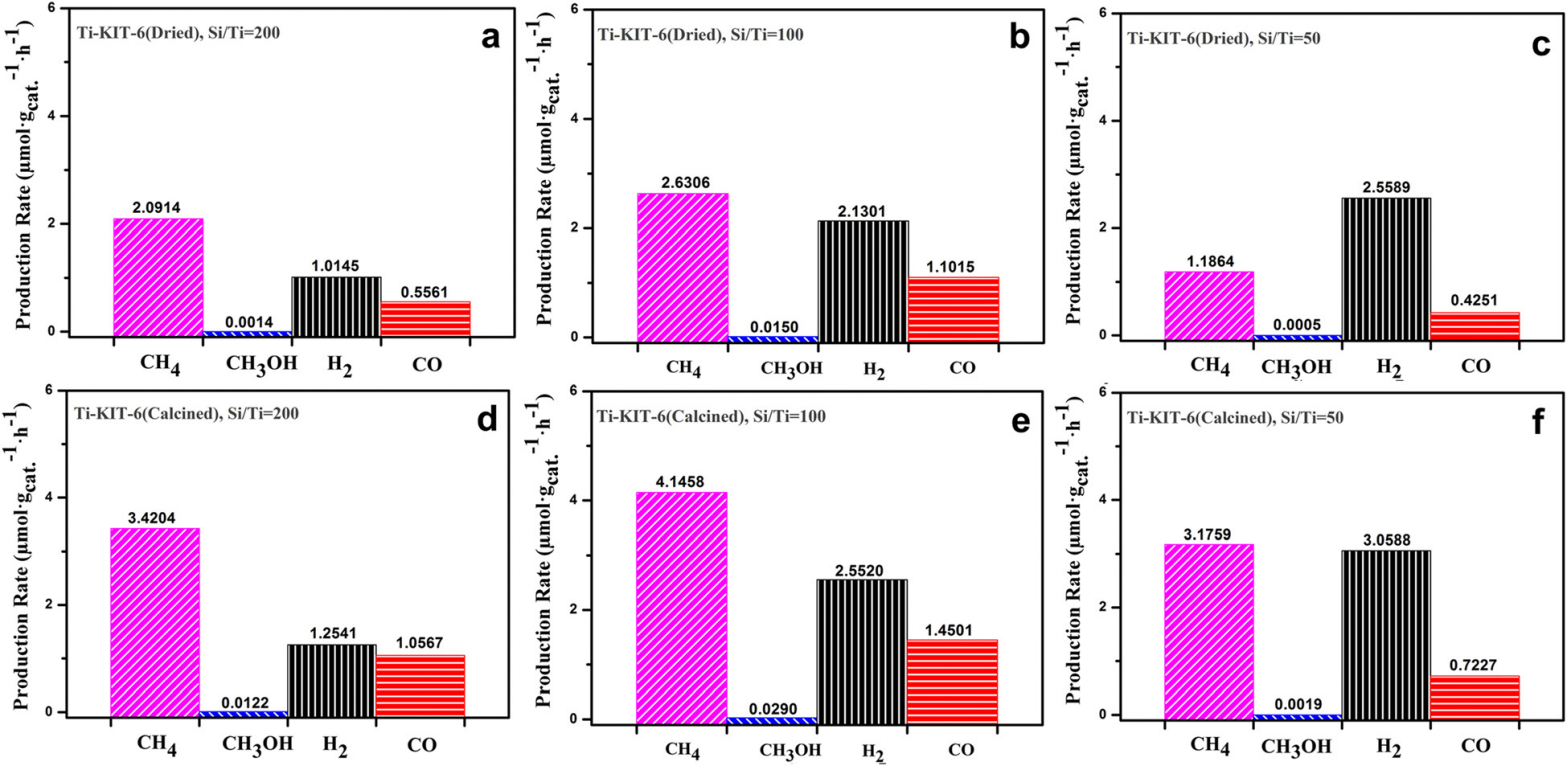
**Comparison of fuel formation after a 3-h photocatalytic reduction of CO**_**2 **_**and H**_**2**_**O vapors. (a-c)** Ti-KIT-6, dried, Si/Ti = 200, 100, and 50 ratios and **(d-f)** Ti-KIT-6, calcined, Si/Ti = 200, 100, and 50 ratios.

The reaction results of the synthesized photocatalysts are shown in Figure 6a,b,c,d,e,f. Blank tests conducted without photocatalysts as well as the reactions in the dark with catalysts have shown no product formation, which indicates that the products obtained during the reaction were merely photocatalyst-based. Figure 6a,b,c shows a comparison of the production rate obtained after 3 h of reaction from the CO_2_ photocatalytic reduction with H_2_O vapors in the presence of Ti-KIT-6 (dried, Si/Ti = 200, 100, and 50 ratios) photocatalysts. As can be seen, CH_4_ was the main product, whereas H_2_, CO, and CH_3_OH (vapors) were also obtained during the reaction when using either Ti-KIT-6 (dried, Si/Ti = 200) or Ti-KIT-6(dried, Si/Ti = 100) materials. However, H_2_ increased and CH_4_ decreased when Ti-KIT-6 (dried, Si/Ti = 50) was used. As already mentioned in the characterization part pertaining to the UV-vis, TEM, and XPS analyses, this phenomenon might be due to the TiO_2_ cluster formation caused by the increased Ti content in the Si/Ti ratio of 50, which favors a greater H_2_ formation [15].

A similar trend of activity was also observed when Ti-KIT-6 (calcined, Si/Ti = 200, 100, and 50 ratios) was used. However, overall, the Ti-KIT-6 (calcined, Si/Ti = 200, 100, and 50 ratios) materials show higher activity than the Ti-KIT-6 (dried, Si/Ti = 200, 100, and 50 ratios) materials. This might be due to the fact that some of the Ti species in Ti-KIT-6 (dried, Si/Ti = 200, 100, and 50 ratios) materials which were not accessible on the surface for the reaction might have been trapped in the bulk dried KIT-6 powder during the synthesis. However, this might not be the problem in the case of Ti-KIT-6 (calcined, Si/Ti = 200, 100, and 50 ratios), where the 3-D pore structure was fully developed in the calcined KIT-6. Therefore, the greater number of accessible active sites in Ti-KIT-6 (calcined, Si/Ti = 200, 100, and 50 ratios) than that in Ti-KIT-6 (dried, Si/Ti = 200, 100, and 50 ratios) may have caused higher activity.

Moreover, it is clear that Ti-KIT-6 (calcined or dried, Si/Ti = 100) shows a higher activity than the Si/Ti ratios of 200 and 50, because of the combined contribution of the high dispersion state of the Ti oxide species, which is due to the large pore size with a 3-D channel structure, and the lower formation of Ti-O-Ti or TiO_2_ agglomerates, as confirmed by UV-vis, TEM, and XPS analyses. Moreover, the high production of CH_4_ for Ti-KIT-6 (Si/Ti = 100) with greater concentrations of the OH groups (2 nm^−1^) than the other ratios (Si/Ti = 200 and 50 = 1.5 and 1.2, respectively) obtained from the FT-IR of the materials actually affects the adsorption properties of the water on the catalyst surface [16]. Competitive adsorption between the H_2_O vapors and CO_2_ is another parameter that can determine the selectivity of CH_4_ or CH_3_OH. CH_4_ formation selectivity becomes higher as H_2_O vapor adsorption increases due to the greater concentration of OH groups or hydrophilicity of the material [4].

The main desired product is CH_4_ as it has a greater energy or heat content [17] than H_2_ or the produced syngas (CO + H_2_), whereas CH_3_OH (vapors) is a minor product. As can be seen in Table 1, it is clear that the abovementioned optimized photocatalysts show more activity than the best commercial TiO_2_ photocatalyst (Aeroxide P25). Moreover, as can be seen in Table 1, the results are comparable with the other results reported in the literature concerning the use of TiO_2_[18], Ti-zeolites or Ti-MCM-41 [16] as a photocatalyst for this application. The optimized Ti-KIT-6 (Si/Ti = 100) showed a relatively better CH_4_ production than the conventional photocatalytic materials, a result that is explained more clearly by examining the reaction mechanism.

**Figure 7:**
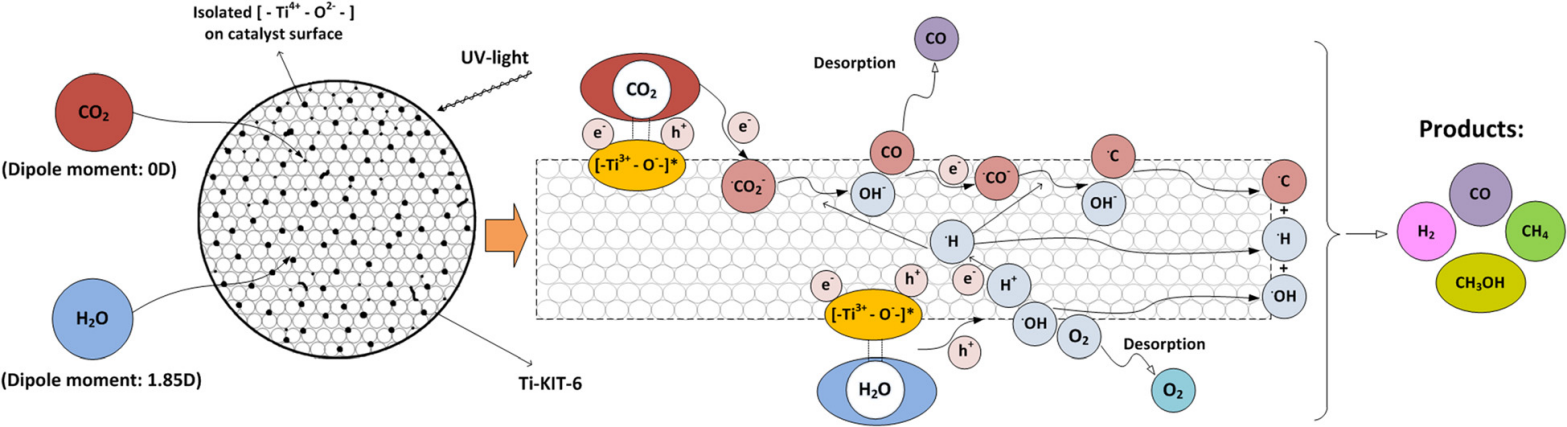
**Reaction mechanism and pathways of the photocatalytic reduction of CO**_**2 **_**with H**_**2**_**O vapor to fuels.**

The CO_2_ photocatalytic reduction mechanism with H_2_O vapors is complex, and two aspects concerning the rate-limiting step should be considered. CO_2_ is a thermodynamically stable compound, and it is difficult to oxidize or reduce it to various intermediate chemicals at lower temperature conditions. Therefore, the first aspect is that the activation of CO_2_ or H_2_O through a charge transfer is the rate-limiting step, whereas the second possibility is that the rate-limiting step in this reaction is the adsorption and desorption of the reactants [19]. Moreover, the carbene pathway has been found to be the most appropriate in the present contest, as CO_2_ photocatalytic reduction active sites are isolated tetrahedrally coordinated Ti^+4^ centers which are embedded in silica or zeolite matrices [20]. The quantum confinement effects in these spatially separated ‘single-site photocatalysts’, upon UV light absorption, cause charge-transfer excited states to be formed. As can be seen in the mechanism shown in Figure 7, these excited states, i.e., (Ti^3+^-O^−^)*, contain the photogenerated electron and hole which are more localized on neighboring atoms [19,20] and are closer than in bulk semiconductors, in which the charge carriers are free to diffuse. Moreover, the lifetime of the excited Ti^3+^-O^−^ is found to be 54 μs [21], which is substantially higher than that of bulk TiO_2_ powder, which is instead of a nanosecond order. Therefore, these active sites in Ti-KIT-6 materials, i.e., (Ti^3+^-O^−^)*, are comparatively more energetic and longer living than those in bulk TiO_2_. Figure 7 shows that CO_2_ and H_2_O are being adsorbed on the surface of the catalyst, with competitive adsorption, due to their different dipole moments. Ti-OH serves as the active sites for the adsorption of the reactants. When the UV light is turned on, the adsorbed CO_2_ and H_2_O vapors interact with the photoexcited active sites, i.e., (Ti^3+^-O^−^)*, inducing the formation of intermediates, including CO, which can be an intermediate as well as a released product, as shown in Figure 7. Finally, C, H, and OH radicals are formed, and these can further combine to form other products, such as CH_4_, H_2_, and CH_3_OH. Therefore, the adsorption and concentration of the OH groups play a key role in this reaction to achieve selective product formation. The optimized photocatalyst has also shown better activity than the best commercial TiO_2_ (Degussa P25, Evonik Industries AG, Essen, Germany), which indicates the importance of future research and further optimization.

## Conclusions

New nanoporous silica (KIT-6 dried or calcined) incorporated with isolated Ti materials with different Si/Ti ratios (Si/Ti = 200, 100, and 50) synthesized has shown that Ti-KIT-6 (calcined, Si/Ti = 200, 100, and 50) were better in activity than the Ti-KIT-6 (dried, Si/Ti = 200, 100, and 50) materials, due to the presence of more accessible surface reaction Ti species. The main fuel products obtained after the reaction are CH_4_, CO, H_2_, and CH_3_OH (vapors). Moreover, it has been found that Ti-KIT-6 (Si/Ti = 100) shows a better product formation than Ti-KIT-6 (Si/Ti = 200 and 50). The high activity of the optimized photocatalyst was found to be due to the lower number of Ti-O-Ti or TiO_2_ agglomerates and to the more isolated Ti species, which were uniformly dispersed on the 3-D KIT-6 mesoporous silica support without damage to mesopore structure. The increased surface concentrations of OH groups found in Ti-KIT-6 also boosted the higher activity. It has been concluded that the activity of the optimized Ti-KIT-6(Si/Ti = 100) is also much higher than that of the commercial Degussa P25 TiO_2_, due to the longer life and the more energetic active sites in the optimized Ti-KIT-6(Si/Ti = 100) photocatalyst than in the bulk commercial TiO_2_ one. These findings indicate that the highly dispersed isolated Ti, within the new KIT-6 mesoporous silica 3-D framework, can be considered a promising and effective photocatalyst for CO_2_ conversion to fuels and as a suitable candidate for other research activities.

## Competing interests

The authors declare that they have no competing interests.

## Authors’ contributions

PA and MH carried out the synthesis of the materials, performed the characterization of the materials, conducted the photocatalytic reaction experiments, and drafted the manuscript. MH, NR, and GS conceived and designed this study. NR and GS also supervised the project, participated in the discussion on the results, and helped improve the manuscript. All authors read and improved the final manuscript.
